# Low prevalence of abdominal aortic aneurysm in the Seychelles population aged 50 to 65 years

**DOI:** 10.5830/CVJA-2012-070

**Published:** 2013-03

**Authors:** Patrick Yerly, George Madeleine, Walter Riesen, Pascal Bovet

**Affiliations:** Department of Internal Medicine, Division of Cardiology, Lausanne University Hospital (CHUV), Lausanne, Switzerland; Unit for Prevention and Control of Cardiovascular Disease, Ministry of Health, Victoria, Seychelles; Institute of Clinical Chemistry and Haematology, Canton Hospital, St Gallen, Switzerland; Institute of Social and Preventive Medicine (IUMSP), Lausanne University Hospital, Lausanne, Switzerland

**Keywords:** abdominal aortic aneurysm, screening, ultrasonography, population-based study, African region

## Abstract

**Abstract:**

The prevalence of abdominal aortic aneurysm (AAA) and its risk factors are well known in Western countries but few data are available from low- and middle-income countries. We are not aware of systematically collected population-based data on AAA in the African region. We evaluated the prevalence of AAA in a population-based cardiovascular survey conducted in the Republic of Seychelles in 2004 (Indian Ocean, African region). Among the 353 participants aged 50 to 64 years and screened with ultrasound, the prevalence of AAA was 0.3% (95% CI: 0–0.9) and the prevalence of ectatic dilatations of the abdominal aorta was 1.5% (95% CI: 0.2–2.8). The prevalence of AAA in the general population seemed lower in Seychelles than in Western countries, despite a high prevalence in Seychelles of risk factors of AAA, such as smoking (in men), high blood pressure and hypercholesterolaemia.

## Abstract

Recent clinical trials have demonstrated a reduction in mortality related to abdominal aortic aneurysm (AAA) in men systematically screened with ultrasound at age 65 to 74 years.^1^-^4^ Risk factors of AAA include male gender, age and smoking, and to a lesser extent hypertension, hypercholesterolaemia and overt atherosclerosis.^5^,^6^ The US Preventive Services task force has recommended the screening of AAA in men aged 65 to 75 years who have ever smoked.^7^

In view of the limited population-based data on AAA available in low- and middle-income countries,^8^,^9^ and none that we are aware of in the African region, we examined the prevalence of AAA in a population-based survey of cardiovascular risk factors conducted in the Republic of Seychelles in 2004. Seychelles is a rapidly developing middle-income island state located in the Indian Ocean approximately 1 800 km east of Kenya (African region).

In 2004, 42.8% of the population was aged less than 25 years and 9.7% was 50 to 64 years old. The majority of the inhabitants is of African descent and a high prevalence of several cardiovascular risk factors was previously demonstrated in the population, particularly high blood pressure.^10^,^11^

## Methods

A random age- and gender-stratified sample of all inhabitants aged 25 to 64 years was drawn using computerised data of a national census carried out in 2002 and thereafter regularly updated by civil status authorities. Methods of the survey have been described previously.^12^

From a total of 1 456 eligible participants (participation rate 80.2%), 566 were aged 50 to 64 years, and 474 took part in the survey (participation rate 83.7%). We restricted the AAA screening to this age range because AAA is rare at younger ages.^13^,^14^

Ultrasound (General Electric LogiqBook connected to a 2–5-MHz transducer, General Electric Health Care, United Kingdom) was performed in the 353 consecutive individuals who took part in the survey during a 17-week period when a sonographer was available. The abdominal aorta was scanned from its most proximal visualisable segment to the iliac bifurcation, both transversally and longitudinally. Its antero–posterior and transverse diameters were measured at their maximal sizes, and the larger of the two values was recorded.

## Results

None of the screened subjects had a history of AAA. The maximal diameter of the aorta could be well visualised in 329 of the 351 eligible participants. AAA, defined as a diameter ≥ 30 mm, was found in only one man (diameter 31 mm, age 59 years, never-smoker, obese, cholesterol 6.7 mmol/l, hypertensive, diabetic). An ectatic dilatation of the aorta (diameter 25–29 mm), which can be regarded as precursor of AAA,^15^ was found in four additional participants: three men and one woman (age: 52, 59, 62 and 63; two ex-smokers; all overweight; three with hypertension; two with diabetes; total cholesterol: 5.0, 6.0, 6.3 and 7.4 mmol/l, respectively).

The prevalence of aneurysm or ectasy of the abdominal aorta of all participants aged 50 to 64 years is shown in Table 1. In the same age category, the prevalence was 15% for current smokers (28% in men, 3% in women), 22% for ex-smokers (32% in men and 3% in women), 70% for overweight participants (body mass index ≥ 25 kg/m^2^), 33% for obesity (≥ 30 kg/m^2^), 70% for high blood pressure (≥ 140/90 mmHg or treatment), 27% for diabetes mellitus and 63% for elevated total cholesterol levels (≥ 5.2 mmol/l).

**Table 1:** Prevalence Of Aneurysm Or Ectasy Of The Abdominal Aorta In The General Population Of Seychelles Aged 50–64 Years

	*Men (n = 151)*		*Women (n = 178)*		*Total (n = 329)*	
	*%*	*95% CI*	*%*	*95% CI*	*%*	*95% CI*
Aneurysm	0.7	0–2.0	0		0.3	0–0.9
Ectasy	2.0	0–4.2	0.6	0–1.7	1.2	0–2.4
Either	2.7	0.1–5.2	0.6	0–1.7	1.5	0.2–2.8

## Discussion

The prevalence of AAA in the general population aged 50 to 64 years seemed lower in Seychelles than in North America or Europe. In North America, in participants aged 50–54/55–59/60–64 years, the prevalence of AAA was 0.9/2.5/4.2% in smokers and 0.2/0.5/0.9% in non-smokers, respectively.^4^,^7^ In Norway, the prevalence of AAA in men/women was 1.9/0% and 6.0/1.1% at ages 45–54 and 55–64 years, respectively.^13^ In the Netherlands the prevalence of AAA in men/women was 0.9/0.2% and 3.1/0.4% at ages 55–59 and 60–64 years, respectively.^16^

In contrast to what was recently described in a population of mainly symptomatic aortic aneurysm patients in Kenya, we did not find a female predominance for the diagnosis of AAA in Seychelles. This was despite the predominant African descent of the population and the prevalence of high blood pressure in the 50- to 64-year age category, which was the leading risk factor associated with aortic aneurysms in this study.^17^ This apparent inconsistency might be due to methodological factors, such as gender differences in health-related habits, since the Kenyan study was based on hospital records and not on population-based data.

A low prevalence of AAA in Seychelles might be consistent with a high prevalence of diabetes and the predominantly African descent of the population, which are two factors reported to be inversely associated with AAA.^6^,^7^ It is however at odds with a high prevalence of smoking (in men), high blood pressure and hypercholesterolaemia in the Seychelles population.

Alternatively, we cannot exclude some imprecision in our estimates in view of the relatively small size of our sample and broad confidence intervals, although the population-based design of the study as well as the high participation rate strengthens the reliability of our epidemiological data. On the other hand, the seemingly higher prevalence of aortic ectatic dilatation could announce increasing rates of AAA in the next decades as the population becomes exposed to high risk-factor levels over long periods of time.

Furthermore, because of a high prevalence of AAA risk factors, such as current smoking (28% in men and 4% in women aged 40–49 years) or high blood pressure (35% in the 40–49- year population) in younger age groups with a lower prevalence of ‘protective factors’ such as diabetes mellitus (11.7% in the 40–49-year population), ectatic lesions might appear at a younger age in Seychelles than in North America or Europe. However, given the small rate of expansion of small lesions over time, the finding of true AAA in subjects aged less than 50 years is unlikely.^18^

## Conclusion

Pending further data on the prevalence of AAA in older age categories, our results do not support routine screening of AAA in the selected population. This is consistent with recommendations for populations in Western countries.^5^
